# Essential newborn care practices and determinants amongst mothers of infants aged 0—6 months in refugee settlements, Adjumani district, west Nile, Uganda

**DOI:** 10.1371/journal.pone.0231970

**Published:** 2020-04-23

**Authors:** Henry Komakech, David Lubogo, Elizabeth Nabiwemba, Christopher Garimoi Orach

**Affiliations:** Department of Community Health and Behavioural Science, Makerere University School of Public Health, Kampala, Uganda; Johns Hopkins School of Public Health, UNITED STATES

## Abstract

**Background:**

Despite recent improvements in child survival, neonatal mortality remains high in most developing countries. Countries affected by humanitarian emergencies continue to report the highest neonatal mortality rates.

**Objective:**

To assess essential newborn care practices and its determinants amongst mothers of infants aged 0–6 months in refugee settlements in Adjumani district.

**Methods:**

A cross-sectional study was conducted among mothers of infants aged 0–6 months in refugee settlements, Adjumani district. A total of 561 mothers of infants were selected using systematic sampling technique from households. Data were collected using a semi-structured questionnaire. A composite outcome variable, Essential Newborn Care practices was created by merging different care practices (neonatal feeding, thermal care, and cord care). Multiple logistic regression analysis was used to determine predictors of Essential Newborn Care.

**Results and conclusions:**

Over half (57%) of the mothers breastfed their newborns within one hour. Half (50.1%) of mothers cleaned the umbilical cord of their newborns. Only 17% of the newborns received optimal thermal care immediately after birth. Mothers aged 20–24 years (OR 0.38, CI 0.17–0.96) and those involved in subsistence farming (OR 0.67, CI 0.38–1.45) were less likely to practice good newborn care compared to those in other occupations. Newborn care practices were sub-optimal in this refugee setting. To improve newborn care practices, there is need to educate mothers through community-based health interventions in order to promote delayed bathing, ideal infant feeding, thermal and umbilical cord care.

## Introduction

Over the last decade, increased commitments towards improving the lives of infants across the globe have resulted in the steady decline of under-five mortality rates. Under-five mortality rates have declined substantially from 12.7 (12.6, 13.0) million in 1990 to 5.9 (5.7, 6.4) million in 2015 [1]. Despite these declines, neonatal mortality rates have remained unacceptably high with an estimated 2.6 million deaths occurring annually. This represents nearly half (46%) of all under-five deaths globally [1]. Unfortunately, majority of newborn deaths are clustered in low-income countries, with two-thirds occurring in Asia and Africa [2]. In 2016, 99% of the 2.6 million newborns deaths occurred in low-income countries [3]. These countries accounted for 22% of the under-five deaths yet they only shared 12% of the under-five population [1]. More than 80% of deaths among children 0–59 months result from premature birth, complications during labour and delivery and infections such as sepsis, meningitis and pneumonia [4].

The World Health Organization (WHO) recommends several strategies aimed at improving maternal and newborn survival [5]. These strategies include the Helping Babies Breathe (HBB) initiative, Essential Newborn Action Plan [6] and the Strategies Toward Ending Preventable Maternal Mortality [7]. The Essential Newborn Care (ENC) practices include a package of interventions such as clean cord care (cutting the umbilical cord with a sterilized instrument and tying it with a sterilized thread), delayed bathing and initiating breast feeding within the first 1 hour after birth [8, 9]. These low cost, high impact interventions are critical for survival and have been demonstrated to save newborn lives in various settings [10–12]. Adherence to optimum ENC practices may reduce the risk of newborn morbidity and mortality [10]. However, it is essential that the interventions are integrated into an all-inclusive newborn care package including skilled delivery care, care seeking, extra care for sick and small babies and resuscitation.

Humanitarian emergencies presents several risks to the health and wellbeing of communities caught up in these areas. Such emergencies may lead to increased morbidity and mortality [13]. Countries affected by humanitarian emergencies have the highest maternal and newborn mortality rates [14]. About 250 million children under the age of five live in these countries [15]. Of the ten countries with the highest under-five mortality rates, eight are classified as fragile and conflict affected. About 42% of neonatal deaths occur in fragile and conflict affected countries [16]. About 60% of preventable maternal deaths [14] and 53% of under-five deaths [17, 18] occurred in these countries. While maternal health indicators are dismal in most refugee settings, in Uganda efforts by the government and partners have led to several improvements. Complete Antenatal Care Delivery (ANC) is at 82% while skilled delivery is high 94% in all refugee settlements in the country [19]. These could be attributed to proximity of health facilities and incentives for mothers to deliver in health facilities [20]. We examined the hypothesize that there is no association between various maternal characteristics and newborn care practices.

Despite of the growing evidence of the effects of humanitarian emergencies on child health [21–23], there is little information on ENC practices among mothers of infants in refugee emergencies. This may be attributed to several factors including, greater emphasis being placed on addressing other urgent needs of the refugees including the provision of safe and clean water, shelter, food and nutrition and health services for infectious diseases. Few studies have examined ENC practices in refugee emergencies. A pre-post intervention study in South Sudan’s displacements camps showed poor maternal knowledge of danger signs, optimal feeding practices, fever, breathing difficulties, reduced activity and convulsions [24]. While a study among refugees in Guinea Bissau found that the use of breast milk substitutes increased the risk of infant mortality due to poor hygiene and sanitation and inappropriate preparation of meals. In the same study, the risk of dying was six fold among children aged 9–20 months who had been weaned early compared to those breastfeeding [25]. This study assessed ENC practices and its determinants among mothers of infants 0–6 months in refugee settlements in Adjumani district, Uganda.

## Methods

### Study design, setting and sample

This was a community based cross-sectional study conducted during October—December 2016 in Adjumani district, west Nile, Uganda. The district has an estimated population of 432,000 inhabitants [26]. These includes an estimated 240,905 refugees living in 17 designated settlements [27]. Health service for refugees and host communities are provided by 42 public health facilities. These include Adjumani district hospital, three Health Centre IV’s and twenty-one Health Centre IIs. Health Centres are part of the district health systems in Uganda. The district health system consists of several levels. Village Health Teams (VHTs) are volunteer community health workers who deliver health education, preventive and simple curative services in communities. The VHTs constitute level one health services. The next are Health Center IIs, that provide outpatient care and are managed by a nurse serving approximately 5,000 people. Health Center IIIs (HC III) serve 10,000 people and provide in addition to HC II services in patient, simple diagnostic and maternal health. It is managed by a clinical officer. Health Center IV’s are managed by a medical doctor and provide surgical services in addition to all other services provided at HC III. Other conditions requiring higher level care are referred and managed at the Adjumani district hospital and Mulago National Referral Hospital.

Health facilities provide a range of services including health promotion, curative, Primary Health Care, delivery and minor procedures. Maternal and child health services include ANC, delivery and postnatal care are provided in several facilities. Utilization of these services by mothers in most facilities is high deliveries by skilled health worker at 93% and complete ANC at 84%. Reasons for these high rates of utilization includes close proximity of health facilities in or around settlements and health education and promotion for mother and newborn care in this setting.

The sample size was calculated using the Kish and Leslie formulae [28]. A total of 561 mothers was the final sample size for the study. The study population consisted of women of reproductive age between 18–49 years of age who had delivered a baby in the last six months prior to the conduct of this study. The six-month limit was set in order to reduce the chances of recall bias by the mothers of infants. Participants were selected if they were the mother of the infant. Mothers who had lived in the sampled refugee settlements for more than one year were included in the study. Mothers who had a still birth and were seriously ill were excluded.

We used the modified WHO, EPI cluster sampling technique [29]. Refugee settlements in the district are divided into zones and blocks. For this study, a zone was designated as a cluster. The sampling interval was determined by dividing the total population by the number of clusters. An identification form was used to identify the clusters that were included in the study. The initial cluster was determined at the centre of each settlement. A total of 11 refugee settlements were purposefully selected for the study. In each zone, a household list was obtained from the local leader (camp commandant). The first respondent in the initial household was randomly selected. The nearest household whose front door was closest to that which was first sampled was visited next. In each homestead, the household head was approached, informed about the study objectives and requested to provide consent. A total of 46 respondents were interviewed in each cluster. In each household, we interviewed one eligible and consenting adult respondent. The respondent was either the mother of the child or a close caretaker.

### Data collection procedure

Data were collected using a semi-structured interview questionnaire adapted from the WHO indicators for assessment of Infant and Young Child Feeding (IYCF) [30] and by reviewing other literature. The questionnaire includes items on respondents and household socio-demographic characteristics, antenatal care, birth preparedness, delivery and immediate newborn care, nutrition, postnatal care for mother and baby, neonatal illness and care seeking. The questionnaire has been field tested and used in several settings. The study questionnaire was translated from English into the local language (Juba Arabic) spoken widely by refugees in the study area. This was to make the questions easily understandable to the study population and it was retranslated back into the English language. The survey was conducted from October to December 2016. The questionnaires were administered to respondents by trained research assistants with public health background, skills in data collection and experience from previous studies. The research assistants and supervisors were recruited and trained for two days on the study objectives, data collection procedures and ethics by the authors. The research assistants approached, screened, selected households and administered the questionnaire to participants.

The dependent variable was newborn care practices. These included cord care, optimal thermal care and breastfeeding initiation. The dependent variable for the study was a binary outcome created by categorizing three variables including good neonatal feeding that is if breastfeeding was initiated within the first hour after delivery and late initiation if initiated more than 1 hour after birth. This includes the proportion of children aged 0–6 months who were exclusively breastfed, while acknowledging that it may be lower than the current status. Good cord care in this study was defined as use of a clean instrument to cut the umbilical cord, clean thread to tie the cord and no substance applied to the cord. Optimal thermal care referred to placing the baby on Skin-to-Skin contact or wrapping at birth plus giving the first bath after 6 or more hours. Independent variables included mother’s socio-demographic characteristics.

### Data management and analysis

Completed questionnaires were checked for completeness and coded. Double data entry was conducted using Epi Data version 3.1. Discrepancies identified in the data were reconciled by referring to the original survey forms. Further data entry was conducted to correct for inconsistencies identified during exploration and analysis and recorded in an analysis log. Data were exported into Stata SE version 14 for analysis. A composite outcome variable was created based on the following variables: (a) Good cord care (b) Thermal care and (c) Good neonatal breastfeeding. The composite variables were dichotomized to Yes (all practices present) or No (one or more practices missing).

Descriptive analysis was conducted to produce tables of frequencies and proportions. Binary logistic regression was conducted to determine the association between the independent and dependent predictors of ENC practices. Variables with a p < value less than or equal to 0.05 were considered significant for the multivariable logistic regression analysis. Back ward logistic regression was used with a p < value of less than 0.05 considered significantly associated with ENC practices. The strength of association between dependent and independent variables was expressed by using odds ratio with a 95% confidence interval.

This study was approved by the Higher Degrees and Research Ethics Committee of Makerere University School of Public Health and Uganda National Council for Science and Technology. Permission to access the refugee settlements and conduct the study was obtained from the Office of the Prime Minister and United Nations High Commission (UNHCR) and the District Health Office and managers of health facilities in Adjumani district. Written informed consent was obtained from all participants prior to conducting the interviews. Participants were informed that participation in the study was voluntary and they could withdraw at any stage of the interview.

## Results

A total of 561 respondents participated in this study. Nearly 60% of the respondents were aged between 20–34 years (Table 1). Majority 95% of the respondents were married with 55.8% having had four or more children. More than half 59.7% of the respondents were in a polygamous marriage. About 54.2% of respondents had no formal education.

**Table 1:** Socio-demographic characteristics of mothers of infants aged 0—6months in refugee settlements Adjumani district *(n = 561)*.

Variables	Total (n)	Percentage (%)
**Respondents age (years)**		
<19	27	4.8
20–24	127	22.6
25–34	205	36.6
30–34	121	21.6
>35	81	14.4
**Relationship to child**		
Mother	550	98.0
Caregiver	11	2.0
**Marital status**		
Married	553	98.6
Not married	8	1.4
**Type of marriage**		
Monogamous	226	40.3
Polygamous	335	59.7
**Religion**		
Catholic	361	64.4
Protestant	187	33.3
Moslem	13	2.3
Pentecostal		
**Educational level**		
None	304	54.2
Primary	181	32.3
Secondary	41	7.3
Tertiary	35	6.2
**Occupation of mother**		
Subsistence farmer	40	7.1
Business	21	3.7
Housewife	500	89.1
**Sex of last child**		
Male	317	56.5
Female	244	43.5
**Parity**		
1	76	13.5
2–3	172	30.7
>4	313	55.8

More than half (57%) of the mothers breastfed their newborns within one hour (Table 2). Majority (94.7%) of mothers gave colostrum to their babies. Half (50.1%) of mothers cleaned the cord of their newborns appropriately. About 12.7% of babies had a bath 24 hours or more after delivery. Only about 17% of the newborns received optimal thermal care immediately after birth.

**Table 2:** Selected newborn care practices by study respondents in refugee settlements Adjumani district *(n = 561)*.

Variables	Total (n)	Percent (%)
**Timely initiation of breastfeeding**		
Yes	319	57.0
No	242	43.0
**Colostrum feeding**		
Yes	529	94.3
No	32	5.7
**Pre-lacteal feeding**		
Yes	54	9.6
No	507	90.4
**Bottle feeding**		
Yes	157	28.0
No	404	72.0
**Mixed feeding**		
Yes	442	78.2
No	119	21.8
**Cord cleaning**		
Water only	227	40.5
Water with soap	178	31.7
Salted water	77	13.7
Disinfectant	26	4.6
Other	52	9.4
**What was put on the cord**		
Nothing	221	39.4
Powder	112	20.0
Ash	67	11.9
Spirit	108	19.3
Herbs	17	3.0
Others	36	6.4
**Baby`s first birth**		
Immediately	201	35.8
1–6 hours	232	41.4
7–12 hours	32	5.7
13–24 hours	25	4.5
After 24 hours	71	12.7
**Kangaroo Mother Care (KMC)**		
Yes	99	17.6
No	462	82.4

Cord care, thermal care and early breastfeeding by the socio-demographic factors are presented in (Table 3). Maternal age (*x*^2^*prob*< 0.030) and maternal occupation (*x*^2^*prob<* 0.014) were associated with safe cord care, while religion (*x*^2^*prob<* 0.000) was an important predictor of early breastfeeding. No significant association was observed between optimal thermal care and any of the socio-demographic variables.

**Table 3:** Selected essential newborn care practices by socio-demographic characteristics of respondents in refugee settlements Adjumani district.

Variables	Thermal care (n = 99)		Safe cord care (n = 322)		Early breastfeeding (n = 319)	
	(% yes)	*p-value*	(% yes)	*p-value*	(% yes)	*p-value*
**Respondents age**		0.903		0.030		0.190
<19	5.0		2.8		6.0	
20–24	19.2		22.4		19.4	
25–34	36.4		40.7		38.2	
30–34	23.2		20.5		21.9	
>35	16.2		13.7		14.4	
**Marital status**		0.187		0.252		0.066
Currently married	100		99.1		99.4	
Not married	0.0		0.9		0.6	
**Type of marriage**				0.129		0.750
Monogamous	45.5		37.6		40.8	
Polygamous	54.5		62.4		59.2	
**Religion**		0.248		0.106		0.000
Protestant	63.6		61.2		71.8	
Catholic	34.3		35.7		26.0	
Moslem	2.0		3.1		2.2	
**Educational level**		0.841		0.074		0.072
None	54.4		53.4		56.7	
Primary	32.0		32		28.5	
Secondary	6.2		6.2		6.9	
Tertiary	8.4		8.4		7.8	
**Occupation**		0.660		0.014		0.010
Housewife	87.9		86.3		90.0	
Subsistence farmer	9.1		8.1		5.3	
Business	3.0		5.6		4.7	
**Sex of last child**		0.051		0.993		0.490
Male	47.5		56.5		55.2	
Female	52.5		43.5		44.8	
**Parity**		0.305		0.147		0.173
1	14.1		11.2		13.8	
2–3	24.2		30.7		27.6	
>4	61.6		58.1		58.6	

The main predictors of essential newborn care in this study included mother’s age, educational level, occupation and religion as indicated in (Table 4). Compared to mothers 30 years and above, those aged 20–24 years (AOR 0.37, 95% CI: 0.16, 0.91) were less likely to have practice good cord care. Mothers who had obtained at least primary education were more likely (AOR 1.45, 95% CI: 3.11, 7.14) to have initiated breastfeeding early as were those involved in subsistence farming (AOR 2.54, CI: 1.12, 5.77). Mothers who are Catholics were almost two times more likely to have breastfed their newborn early compared to those of other religions (AOR 1.89, CI: 1.10, 2.23).

**Table 4:** Logistic regression model for essential newborn care practices (cord care and earl breastfeeding) by socio-demographic factors in refugee settlements, Adjumani district.

Variables	Cord care				Early breastfeeding			
	Univariate		Multivariate*		Univariate		Multivariate*	
	OR	95% CI	OR	95% CI	OR	95% CI	OR	95% CI
**Mothers age**								
<19	1	1	1	1	1	1	1	1
20–24	0.37	(0.16,0.91)	0.38	(0.15,0.93)	1.07	(0.29,3.99)	1.03	(0.27,3.90)
25–34	0.31	(0.12,0.66)	0.31	(0.13,0.76)	0.81	(0.26,2.97)	0.86	(0.23,3.27)
30–34	0.42	(0.17,1.00)	0.43	(0.17,1.06)	1.13	(0.30,4.22)	1.20	(0.31,4.69)
>35	0.42	(0.17,1.05)	0.42	(0.16,1.10))	1.13	(0.29,4.43)	1.27	(0.30,5.32)
**Educational level**								
None	1	1	1	1	1	1	1	1
Primary	0.99	(0.68,1.43)	1.04	(0.16,1.55)	1.45	(3.11,7.40)	1.36	(0.72,2,55)
Secondary	1.37	(0.71,2.63)	1.54	(0.76,3.11)	1.62	(0.81,2.58)	1.48	(0.54,4.06)
Tertiary	0.39	(0.17,0.89)	0.55	(0.23,1.30)	0.89	(0.26,3.08)	0.92	(0.24,3.40)
**Occupation**								
Housewife	1	1	1	1	1	1		1
Subsistence farmer	0.67	(0.34,1.32)	0.82	(0.41,1.66)	2.93	(1.35,6.35)	2.54	(1.12,5.77)
Business	0.21	(0.06,0.72)	0.29	(0.08,1.07)	0.44	(0.06,3.35)	0.45	(0.55,3.63)
**Religion**								
Protestant	1	1	1	1	1	1	1	1
Catholic	0.75	(0.52,1.08)	0.75	(0.51,1.10)	1.89	(1.10,3.23)	1.55	(0.88,2.77)
Moslem	0.36	(0.97,1.33)	0.29	(0.08,1.12)	0.85	(0.11,6.80)	0.85	(0.10,6.93)

Adjusted for mothers age, education, occupation and religion

* *p<* value for the model is 0.0051

## Discussions

This study was one of the few studies that has assessed the patterns and determinants of newborn care practices among mothers of infants in refugee settings. The findings are drawn from a sample of mothers of infants living in refugee settlements indicate sub-optimal newborn care practices, early breast-feeding initiation 57.0%, thermal care at 17.4%, and cord care at 39.4%. To the knowledge of the authors, no studies have evaluated newborn care practices among mothers of infants in refugee settlements in Uganda or similar settings. The study adds to a small but growing literature on newborn care practices among refugees and other displaced populations [24, 31, 32].

In this study, 57% of the mothers-initiated breastfeeding within one hour after birth. This finding falls short of the recommended practices and puts infants at risk of increased morbidity and mortality. The finding is much lower than (97.7%) of mothers initiating breastfeeding within one hour in a health facility based study among encamped South Sudanese refugees [33]. While a study among camp based Saharawi refugees in Algeria found breastfeeding initiation to be about 64.9% [34]. These studies findings are well above the international rates of 40%. However, they remain far from optimal. Early initiation of breastfeeding is encouraged as mothers benefit from early suckling because it stimulates breast milk production and facilitates the release of oxytocin, which helps the contraction of the uterus and reduces postpartum blood loss. Further, the Interagency Field Manual outlines the care to be provided during pregnancy and childbirth and these include early and exclusive breast feeding, maternal nutrition during antenatal care, labour and delivery and postnatal care [35].

In humanitarian emergencies settings, infant feeding is an important determinant of morbidity and mortality. Factors related to inadequate access to food, poor feeding practices, and vulnerability to infections [36] may lead to increased risks among infants. In many humanitarian emergencies, the physical conditions in which displaced populations live are highly pathogenic settings characterised by congestion, poor sanitation and hygiene [37]. The risk of infection highlights the importance of early and exclusive breastfeeding as it is a safer and sustainable infant feeding option in the face of inadequate access to safe water and sanitation facilities and poverty [38]. A study among infants in Kurdish refugees found that an estimated 12% died in the first 12 months of the emergency due to high rates of formula feeding in a food insecure and unhygienic environment [39]. Mortality among children under five years was above the international threshold following floods in Botswana in 2006, with an estimated 500 deaths due to diarrhoea [40]. This was attributed to suboptimal breastfeeding as infants not breastfed were 30 times or more likely to require treatment for diarrhoea than those who received breast milk. In the same study, 27 out of the 131 children under two years who were hospitalized and not breastfed, 27 died [40] [41].

Appropriate cord care is essential for infant survival and a means of ensuring an event-free first few weeks of life. This study observed inadequate cord care practices by mothers. A third of mothers used only water to clean the cord while more than half used some form of disinfectant and other substances to clean the baby’s cord. Mothers may have applied these substances to the cord stump in order to accelerate the healing process, prevent inflammation and infections. The use of plain water to clean the babies cord was reported in a study conducted in similar rural settings in Uganda [42]. While a study in rural areas of Ghana found poor cord care practices by mothers of infants [43]. The WHO guideline for the use of antiseptics indicate that harmful, unhygienic, traditional practices places newborn at increased risk for omphalitis [44]. Challenges in cord care by mothers highlight the need to guarantee safe and favourable cord care practices in order to reduce morbidity and mortality related to infections. Owing to the study design used, we did not assess the outcome of cord care as an important determinant between cord separation and sepsis within the neonatal period. Further studies should be conducted to establish this relationship within the neonatal period.

Early Skin-to-Skin Contact (SSC), is an essential physiological norm and is highly recommended as standard practice and as part of UNICEF baby friendly health initiative [45]. In this study, SSC was practiced by a small proportion of mothers. While we did not directly assess exactly how mothers applied SSC but overall, newborn warm practice was poor. This finding is similar to a study by [46] among Karen refugees that found poor SSC despite of high rates of breast feeding initiation by mothers. Other studies have established poor SSC practices by mothers in various rural African settings [47, 48]. [49]. According to the WHO, SSC is a high-impact intervention that supports the establishment of exclusive breastfeeding, assists with appropriate thermal care and promotes bonding between mother and newborn [50].

In this study, maternal age was significantly associated with newborn care practices. Mothers aged 20–24 years were less likely to practice good newborn care compared to older ones. This may be explained by most women in rural settings getting married off at a young age. Coupled with low levels of education, these young mothers may not be adequately prepared for childcare. Migrant and refugee adolescent and young women may face greater challenges in childcare. In many low- and middle-income settings, young women often get married off early and are initiated into family life and childbearing in these settings. This has several consequences with a wide range of health-related, social and economic challenges including financial dependency, gender inequality and increased risks of negative health outcomes for mother and newborn. These factors have been cited as reasons for poor newborn outcomes.

Findings of this study have important implications for policy and programmes if newborn care and practice is to be improved in refugee emergencies and similar settings. Based on the evidence of this study, ENC practices were inadequate among mothers. In order to ensure effectiveness of newborn care interventions, programmes implementation should focus on community-based interventions that target the vulnerable members of the community while utilising existing structures and networks. Finally, we recommend further evaluation of newborn care practices should include both the individual but also the community practices.

The findings from this study may not be generalizable to other refugee settings as it was conducted among households in unique refugee settlements where health facilities are easily accessible. Further, this study highlights some of the difficulties in measuring newborn care practices in certain settings especially among displaced populations. First, it is uncertain how mothers or caretakers accurately recall time, actions and support especially immediately and after delivery. Participants may instead have provided responses to in on order to ensure they complete the interview. Finally, the study questionnaire was translated and back translated a priori and administered in languages spoken by the refugees. This limitation raises concern over the validity of the reported frequencies of essential newborn care practices in this setting.

Despite the challenges and limitations, this study helps to illuminate the relationship between population displacement and newborn care in low-and-middle income countries. While the study utilized a cross-sectional design, it provides important baseline information on critical newborn practices among refugees caught up in protracted displacements in low-and-middle-income countries. The findings of this study may therefore contribute to the identification and implementation of initiatives aimed at improving maternal and newborn care practices that are important towards setting a healthier life-course.

## Conclusion

Essential newborn care practices in this refugee setting were generally inadequate. This finding may suggest that ENC interventions are not accessible and being utilized by mothers in this setting. Poor and high-risk newborn care practices point to the need for an urgent practical intervention in refugee settings. It is therefore important to promote and facilitate health facility deliveries, delayed bathing for 24 hours by mothers and immediate and exclusive breastfeeding in this study population. District health services, aid agencies and other stakeholders should strengthen community-based newborn care interventions. This could be enhanced by establishing or strengthening linkages between health facilities and communities’ in order to reduce risk factors that contribute to newborn morbidity and mortality. Finally, further studies should be conducted on the risk of newborn mortality and factors associated with sub-optimal newborn care practices in other refugee settings.

## Supporting information

S1 File (DTA)

## Abbreviations

ENAP: Essential Newborn Action Plan
ENC: Essential Newborn Care
HBB: Help Babies Breath
IYCF: Infant and Young Child Feeding
NGO: Non-Governmental Organisations
SSC: Skin-to-Skin Contact
UNHCR: United Nations High Commission for Refugees (UNHCR)
UNICEF: United Nations Children Emergency Fund
WHO: World Health organization
